# Nitrogen Mediates Amino Acid Metabolism Affecting Stachydrine Synthesis in *Leonurus japonicus*

**DOI:** 10.3390/plants15182767

**Published:** 2026-09-10

**Authors:** Xiao-Hong Song, Rui-Xue Wang, Jian-Cai Xiao, Jun-Ling Hou, Bin-Bin Yan, Shou-Dong Zhu, Chao-Geng Lv, Yi-Heng Wang, Yu-Fei Zhang, Jia-Yin Xu, Lei Zhang, Ying Li, Wen-Quan Wang, Yan Zhang

**Affiliations:** 1 National Key Laboratory for Quality Ensurance and Sustainable Use of Dao-di Herbs, National Resource Center for Chinese Materia Medica, China Academy of Chinese Medical Sciences, Beijing 100700, China; sxhlumcl@163.com (X.-H.S.); wangruixue330@163.com (R.-X.W.); xjc1770557949@163.com (J.-C.X.); yb51598@126.com (B.-B.Y.); seaky2002@163.com (S.-D.Z.); lcgfim@126.com (C.-G.L.); wyh-wyh@nrc.ac.cn (Y.-H.W.); 2 School of Chinese Pharmacy, Beijing University of Chinese Medicine, Beijing 100102, China; mshjl@126.com; 3 Tianjin Research Institute of Construction Machinery Co., Ltd., Tianjin 300409, China; zhangyufei@sinomast.com.cn (Y.-F.Z.); tgyznzb@126.com (J.-Y.X.); 13512005456@126.com (L.Z.)

**Keywords:** *Leonurus japonicus*, stachydrine, amino acids, nitrogen, metabolism

## Abstract

*Leonurus japonicus* is a vital herb known for its blood-activating and menstruation-regulating properties, with stachydrine being a crucial secondary metabolite contributing to these effects. Enhancing the accumulation of active ingredients is a key concern in cultivation. This study utilized a hydroponic experiment with varying inorganic nitrogen (N) concentrations (supplied as Ca(NO_3_)_2_·4H_2_O and KNO_3_) as nutrient substrates, demonstrating that exogenous nitrogen input significantly increased biomass and promoted stachyridine synthesis in *L. japonicus* over an extended incubation period. Analysis of key intermediates in the stachydrine synthesis pathway suggests that exogenous nitrogen enhanced the amino acid synthesis pathway in primary metabolism, promoting glycolysis and accumulating tricarboxylic acid (TCA) cycle substances, namely, pyruvate and α-ketoglutarate. This, in turn, enhanced the production of stachydrine precursors—glutamic acid and ornithine. Nitrogen’s involvement redirected the amino acid synthesis pathway more towards the alkaloid synthesis pathway. The study confirmed the essential role of nitrogen in promoting the quality formation of *L. japonicus* and provided initial evidence for the mechanism behind stachydrine formation, offering theoretical and practical insights for improving *L. japonicus* cultivation technology.

## 1. Introduction

Enhancing the quality of medicinal plants and elucidating the process of medicinal plant quality formation is a focal point in cultivation scientific research [1]. Providing appropriate nutrients or nutritional elements is an indispensable step in promoting the quality formation of medicinal plants [2]. Plants absorb nutrients and essential elements from the soil through their root systems, utilizing them for cell construction, biosynthesis of biomolecules, and sustaining vital life processes [3,4]. However, the nutritional requirements of medicinal plants vary, and understanding the specific demands of elements based on the characteristics and active components of medicinal plants is a key research task [5]. As in our previous studies, nitrogen (N) is a crucial nutrient element involved in the synthesis of amino acids, proteins, and nucleic acids [6,7], influencing the growth rate of plants and the formation of chlorophyll [8]. Potassium, on the other hand, is critically involved in plant osmotic regulation, photosynthesis, enzyme activity, and stress resistance [9]. The unique regulatory mechanisms and functional characteristics among nutritional elements highlight the complexity of their interactions.

*Leonurus japonicus*, renowned for its blood-activating and menstruation-regulating properties [10], has been utilized for thousands of years, with its dried aboveground parts being the primary medicinal components. The herb contains valuable alkaloids, with the highest activity attributed to stachydrine [11]. Reported pharmacological effects of stachydrine include alleviating uterine smooth muscle spasms, regulating endocrine functions, and enhancing blood circulation, aiding in menstrual regulation and pain relief for women [12,13]. Due to its distinctive pharmacological activity, various strategies for increasing the synthesis and accumulation of stachydrine have been explored by pharmacologists [14]. These approaches encompass genetic enhancements, cultivation and management techniques, as well as hormone treatments [15,16]. Therefore, returning to the origin, unraveling the synthesis mechanism of stachydrine, particularly mediating this reaction, is crucial for expanding the production of active constituents. Previous research has suggested the importance of nitrogen in alkaloid synthesis, given that amino acids serve as precursors for alkaloid synthesis, and nitrogen metabolism influences amino acid synthesis in medicinal plants, establishing an interconnected relationship [17,18]. Exogenous nitrogen application may enhance the synthesis and accumulation of nitrogen-containing alkaloids. However, due to the diversion and transfer of secondary metabolites to various metabolic pathways during secondary metabolism in medicinal plants [19,20,21], further research is needed to clarify this possibility. Following photosynthesis, plants undergo carbon metabolism, and the C/N balance in plants strongly influences the allocation of primary and secondary metabolism resources [21,22]. Nitrogen-containing compounds, such as alkaloids, show a negative correlation with plant C/N balance. We hypothesize that exogenous nitrogen input could prompt plants to prioritize nitrogen metabolism, prompting metabolic pathways towards amino acid synthesis. To better demonstrate this hypothesis and elucidate the specific mechanism of nitrogen involvement, we dissected key pathways in amino acid synthesis, namely glycolysis and the tricarboxylic acid (TCA) cycle [23,24]. Our analysis focused on the crucial intermediates in amino acid synthesis, specifically pyruvic acid and α-ketoglutarate. These metabolites are essential precursors for stachydrine synthesis. By monitoring the dynamic changes in the accumulation of these important substances, as well as total alkaloids and stachydrine, through hydroponic experiments with varying nitrogen concentrations and monitoring periods, we aim to validate the effectiveness of nitrogen in promoting *L. japonicus* growth and enhance our understanding of nitrogen’s mediating role in alkaloid synthesis. This study provides valuable insights for the targeted cultivation of medicinal plants.

## 2. Results

### 2.1. Effect of N Concentration on the Growth of L. japonicus

**Figure 1:**
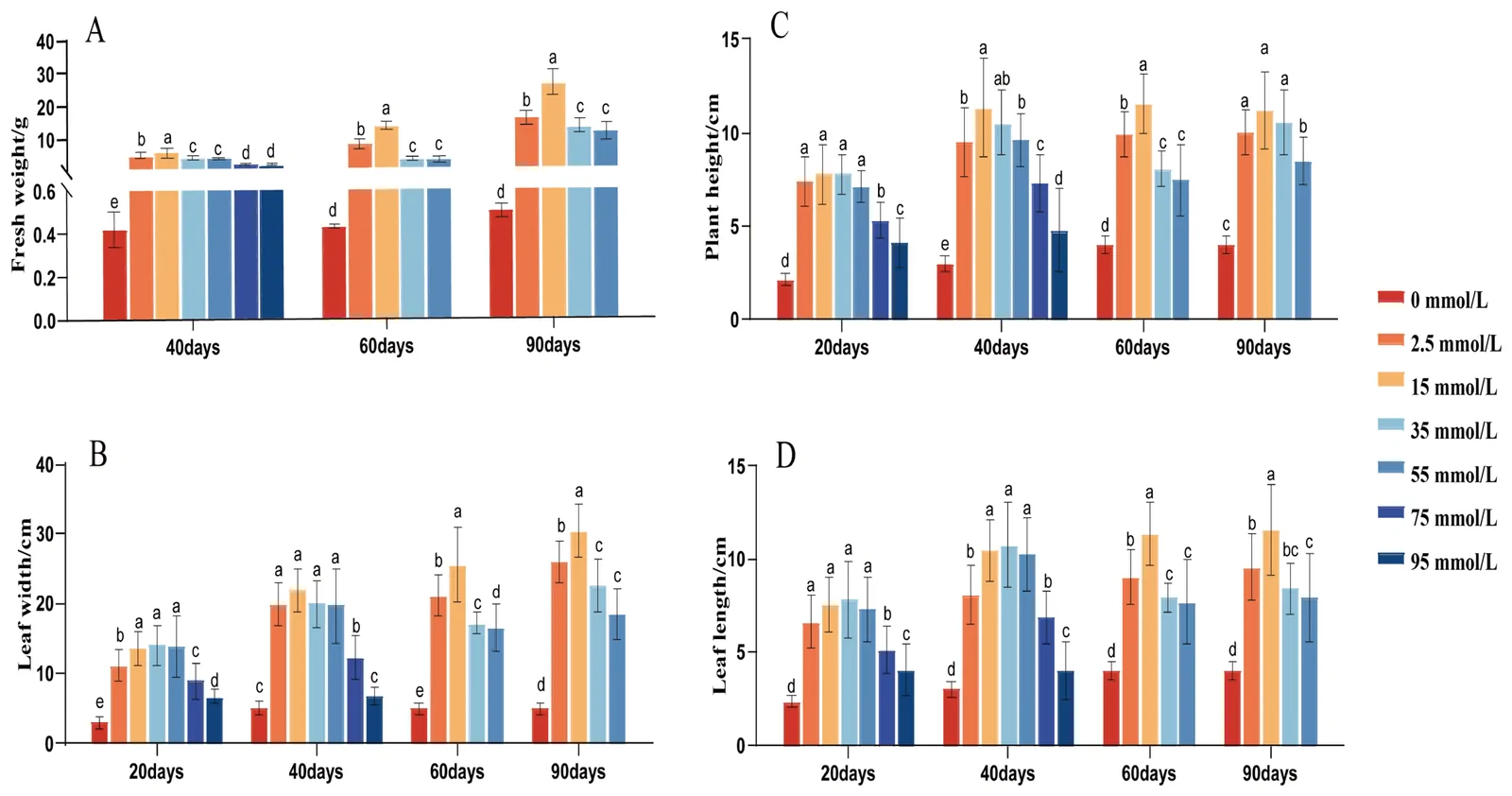
Effect of the application of different concentrations of N on the growth of *L. japonicus.* Effects of different N concentrations on the growth of *L. japonicus* under hydroponic conditions. (**A**) Aboveground fresh weight, (**B**) plant height, (**C**) leaf width, and (**D**) leaf length. Data are mean ± SD (n = 3 biological replicates, with 5 plants pooled per replicate). For each sampling time point (20, 40, 60, and 90 days), bars with different lowercase letters indicate significant differences among N treatments (one-way ANOVA, *p* < 0.05). N0–N7 correspond to N concentrations of 0, 2.5, 15, 35, 55, 75, and 95 mmol·L^−1^, respectively.

The primary medicinal component of *L. japonicus* is the dry aboveground portion, with a specific focus on the leaf yield and quality. To assess these aspects, we conducted measurements of aboveground agronomic traits of *L. japonicus* under varying N concentration treatments and different growth cycles. The results indicated a significant improvement in all growth and development indices compared to the control treatment, irrespective of N concentration (*p* ≤ 0.05) (Figure 1). Interestingly, we observed a pattern in the growth of *L. japonicus* characterized by an initial promotion followed by inhibition with increasing N concentration. Specifically, the growth reached its peak at 15 mmol/L, after which it declined, reaching the lowest point at N concentrations of 95 mmol/L. At excessively high N concentrations (≥75 mmol/L), the growth of *L. japonicus* was hindered, potentially leading to wilting or even plant death due to reverse osmosis of intracellular water and the accumulation of excessive ammonia. Consistent with the general growth and development pattern of plants, we observed overall improvement with cultivation time. However, this trend of enhancement was most pronounced during the 20–40 days stage.

### 2.2. Effect of N Concentration on the Content of Pyruvate Acid and α-Ketoglutarate Acid

**Figure 2:**
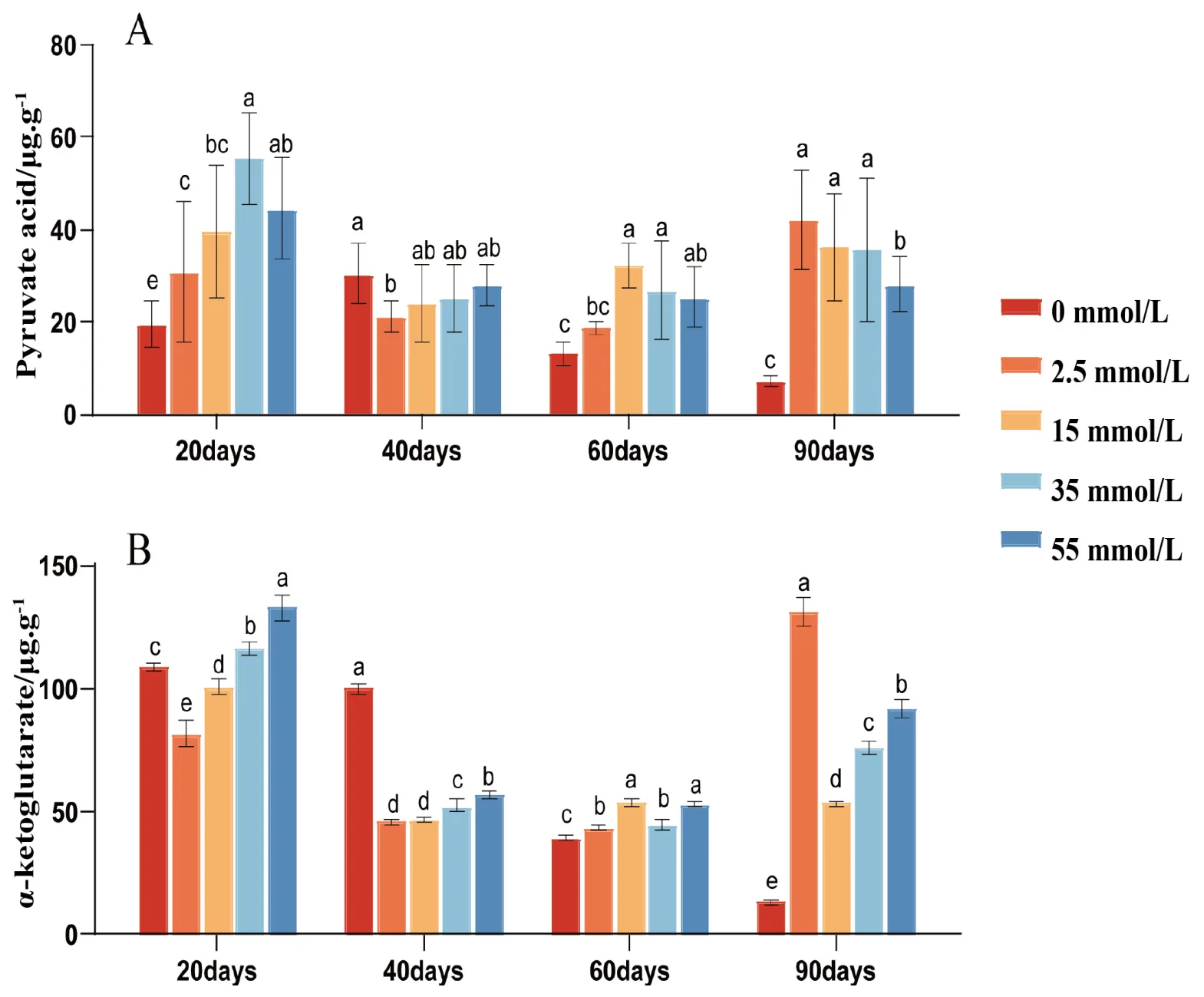
The effect of the application of different N levels on the content of pyruvic acid and α-ketoglutarate of *L. japonicus.* Effects of different N concentrations on pyruvic acid and α-ketoglutarate contents in *L. japonicus* under hydroponic culture. (**A**) Pyruvic acid content; (**B**) α-ketoglutarate content. Data are mean ± SD (n = 3 biological replicates, each replicate consisting of 5 pooled plants). Different lowercase letters above the bars indicate significant differences among N treatments at the same time point (one-way ANOVA, *p* < 0.05). N0–N7 correspond to N concentrations of 0, 2.5, 15, 35, 55, 75, and 95 mmol·L^−1^, respectively.

Pyruvate and α-ketoglutarate serve as crucial precursors in the synthesis of various amino acids for primary metabolism. Moreover, they play a direct role in the tricarboxylic acid cycle, providing both carbon skeletons and energy essential for amino acid synthesis. In our study, the application of exogenous N led to a significant increase in pyruvate content across all stages, excluding day 40. The pyruvate content exhibited a general elevation in the medium- and high-nitrogen concentration treatments (15–35 mmol/L) compared to the low nitrogen treatments (<15 mmol/L), except for a peculiar observation where the pyruvate content peaked at day 20, experienced a decline to a trough at day 40, and thereafter displayed a gradual accumulation trend (Figure 2A). This intriguing pattern was consistently mirrored in the accumulation of α-ketoglutarate content. Notably, α-ketoglutarate levels were initially higher under the control treatment than under N treatment during the early growth period (20–40 days). However, this trend reversed with the prolongation of the growth cycle, with the high nitrogen treatment appearing to be more conducive to the accumulation of α-ketoglutarate (Figure 2B). In summary, our findings underscore the importance of elemental nitrogen in facilitating the accumulation of amino acid precursors, highlighting the dynamic interplay of these metabolites during different stages of plant growth.

### 2.3. Effect of N Concentration on the Content of Amino Acids

**Figure 3:**
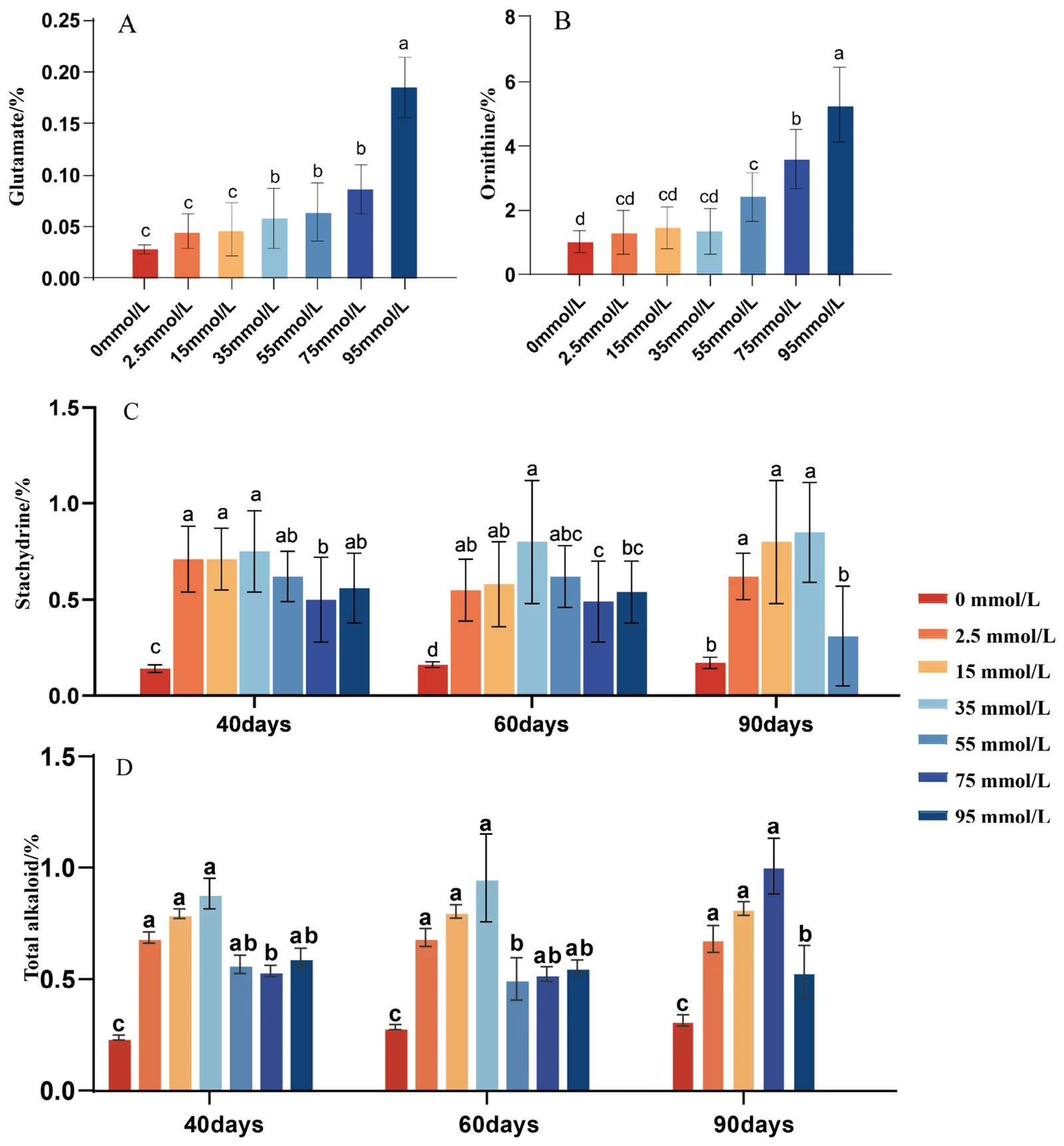
The effect of different nitrogen levels on the content of different amino acids and the content of total alkaloids (**D**) in *L. japonicus* under hydroponic conditions. Effects of different N concentrations on the contents of key amino acids and alkaloids in *L. japonicus* under hydroponic culture. (**A**) Glutamate content; (**B**) Ornithine content; (**C**) Stachydrine content; (**D**) Total alkaloid content. Data are mean ± SD (n = 3 biological replicates, with 5 plants pooled per replicate). For each sampling time point (40, 60, and 90 days), columns with different lowercase letters indicate significant differences among N treatments at the same time point (one-way ANOVA, *p* < 0.05). N0–N7 correspond to N concentrations of 0, 2.5, 15, 35, 55, 75, and 95 mmol·L^−1^, respectively.

In this study, we conducted a comprehensive analysis of 19 amino acids in the leaves of *L. japonicus* on day 60 of the growth cycle (Table 1), taking into account variations in leaf growth and laboratory conditions. Amino acid contents exhibited a progressive increase with higher levels of nitrogen application, demonstrating a dose-dependent response. Remarkably, even under conditions of severe “nitrogen toxicity” (≥75 mmol/L), highly significant differences (*p* ≤ 0.01) were observed when compared to the control. Under nitrogen-deficient conditions, cysteine was nearly undetectable. Notably, glutamate and ornithine emerged as focal points due to their crucial roles as important precursors for stachydrine biosynthesis. Glutamate, in particular, exhibited a fivefold increase in abundance under high nitrogen treatment compared to conditions without nitrogen. Similarly, ornithine showed a sixfold increase in abundance under high nitrogen treatment compared to nitrogen-deficient conditions (Figure 3A,B). These findings underscore the significant influence of nitrogen in the synthesis and accumulation of key intermediates in the alkaloid metabolism of *L. japonicus*. Furthermore, it is crucial to note that the conversion of α-ketoglutarate to glutamate involves a reductive amination reaction, signifying the involvement of nitrogen in the process. Upon comparing the content accumulation trends of α-ketoglutarate and glutamate, we observed that nitrogen element deficiency hindered the conversion of α-ketoglutarate to glutamate, resulting in a transient increase in the substrate’s content (Figure 2 and Figure 3). Thus, the reductive amination reaction can tentatively be regarded as a key rate-limiting step in amino acid synthesis.

### 2.4. Effect of N Concentration on the Content of Stachydrine and Total Alkaloid

We observed a distinctive trend in the stachydrine content, which exhibited an initial increase followed by a decrease with rising N concentration, reaching its peak under N4. This pattern became more pronounced with longer incubation times (Figure 3C). Notably, in the N-free treatment, the stachydrine content consistently remained significantly lower than that observed in the other N-applied treatments, highlighting the robust influence of nitrogen on stachydrine synthesis. Unfortunately, 90 days later, plants subjected to the N6 and N7 treatments succumbed to high N poisoning. However, with the extension of the incubation time, while the accumulation of stachydrine content increased in the low and medium nitrogen concentration treatments (≤35 mmol/L), no significant differences were observed among the groups; the incubation time appeared to have a lesser effect on the synthesis of stachydrine during this extended period. These observed patterns were consistent with the accumulation of total alkaloid content (Figure 3D).

## 3. Discussion

### 3.1. N Significantly Improved the Yield of L. japonicus

Nitrogen (N) serves as a pivotal limiting factor in plant growth, development, and yield formation [25], ranking as the fourth most crucial element in plants, following carbon, oxygen, and hydrogen [26]. Photosynthesis represents a pivotal process through which plants acquire energy and a carbon source. Chlorophyll, serving as a key pigment facilitating photosynthesis, involves N as a fundamental component within its molecular structure [27]. Nitrogen is integral to both photosynthesis and chlorophyll synthesis, contributing significantly to the safeguarding of normal plant growth and development. Moreover, it forms the foundation for alkaloid synthesis in plants [18]. A plethora of studies underscores the significance of nitrogen for the growth of medicinal plants, particularly root herbs like Astragalus membranaceus [28,29]. Nitrogen application proves advantageous in enhancing Astragalus membranaceus yield and promoting the accumulation of Astragalus methylside [30]. However, it is important to note that nitrogen requirements vary among plants. For instance, *Medicago sativa* L. exhibits a conservative approach in nitrogen utilization, with nitrogen application not resulting in increased plant yield [31,32]. Consequently, conducting a preliminary assessment of nitrogen preferences in plants becomes imperative. In our investigation, we observed that nitrogen is indispensable for the growth of the above-ground parts (medicinal portions) of *L. japonicus*. Nevertheless, there is a concern regarding the control of nitrogen concentration in crop cultivation. The growth of *L. japonicus* can be negatively impacted, leading to potential lethality when the nitrogen concentration surpasses the critical value (>55 mmol/L). This phenomenon is more pronounced in grass plants, where doubling nitrogen application results in a reduction in wheat yield.

### 3.2. Mechanism of Action of N Involved in the Synthesis of Stachydrine

N plays a crucial role in both the primary and secondary metabolism of plants. It serves as an indispensable component in the composition of amino acids, which serve as the fundamental building blocks of proteins [33]. Proteins, in turn, are key participants in various biochemical processes within plant cells [34]. The uptake of nitrogen by plants, its synthesis into amino acids, and subsequent protein synthesis are pivotal for supporting proper cell division, growth, and overall cellular function. The synthesis of alkaloids, vital compounds with diverse roles in plant defense against environmental stresses, natural enemies, or pathogens, typically involves amino acids as precursors [35]. Amino acids, being the organic form of nitrogen, are synthesized by plants through the uptake and conversion of inorganic nitrogen [36]. Specific amino acids may serve as direct precursors to alkaloids or undergo a series of enzyme-catalyzed and metabolic pathways leading to alkaloid formation [37]. The level of nitrogen supply profoundly influences the synthesis of secondary metabolites in plants. Optimal nitrogen supply is crucial for promoting the synthesis of secondary metabolites, thereby enhancing plant adaptability to external stresses [38]. In our study, we observed a consistently positive impact of moderate nitrogen levels on the synthesis of both amino acids and total alkaloids. These findings align with previous studies on herbs such as Ligusticum chuanxiong [39] and Sophora alopecuroides [40].

In our discussion, we aim to emphasize the pivotal mediator role played by nitrogen elements in primary metabolism. Previous studies have elucidated that following photosynthesis, plants convert carbon dioxide and water into glucose and oxygen [41]. Subsequently, a glycolytic pathway ensues, breaking down glucose into simpler compounds to generate energy [24]. This process is crucial for maintaining plant energy supply during periods when photosynthesis is not occurring. Within this glycolytic pathway, glucose undergoes conversion to pyruvate, which enters the tricarboxylic acid cycle [23]. Subsequently, pyruvate is transformed into glutamate through the reductive amination of α-ketoglutarate catalyzed by the enzyme glutamate synthase. The synthesized glutamate is then further transformed into ornithine through a five-step enzyme-catalyzed cycle involving Arg J, Arg B, Arg C, and Arg D [42]. Ornithine serves as a crucial building block for subsequent alkaloid synthesis. Throughout this process, the significance of pyruvate and α-ketoglutarate becomes evident. Our results clearly demonstrate the positive impact of appropriate nitrogen concentration on the accumulation of these two substances (Figure 2). Notably, the reductive amination of α-ketoglutarate to glutamate represents a rate-limiting step influenced by nitrogen availability. This step primarily involves the reaction of ammonia (NH_3_) with α-ketoglutarate to form glutamate, a principal nitrogen storage form [43]. Based on our findings, we propose a hypothesis that exogenous nitrogen input contributes substantially to increasing the rate of glutamate synthesis, along with subsequent accumulation of this substance, particularly under conditions of plant nitrogen deficiency. Our significant results provide valuable insights into justifying the dynamics of this process.

**Figure 4:**
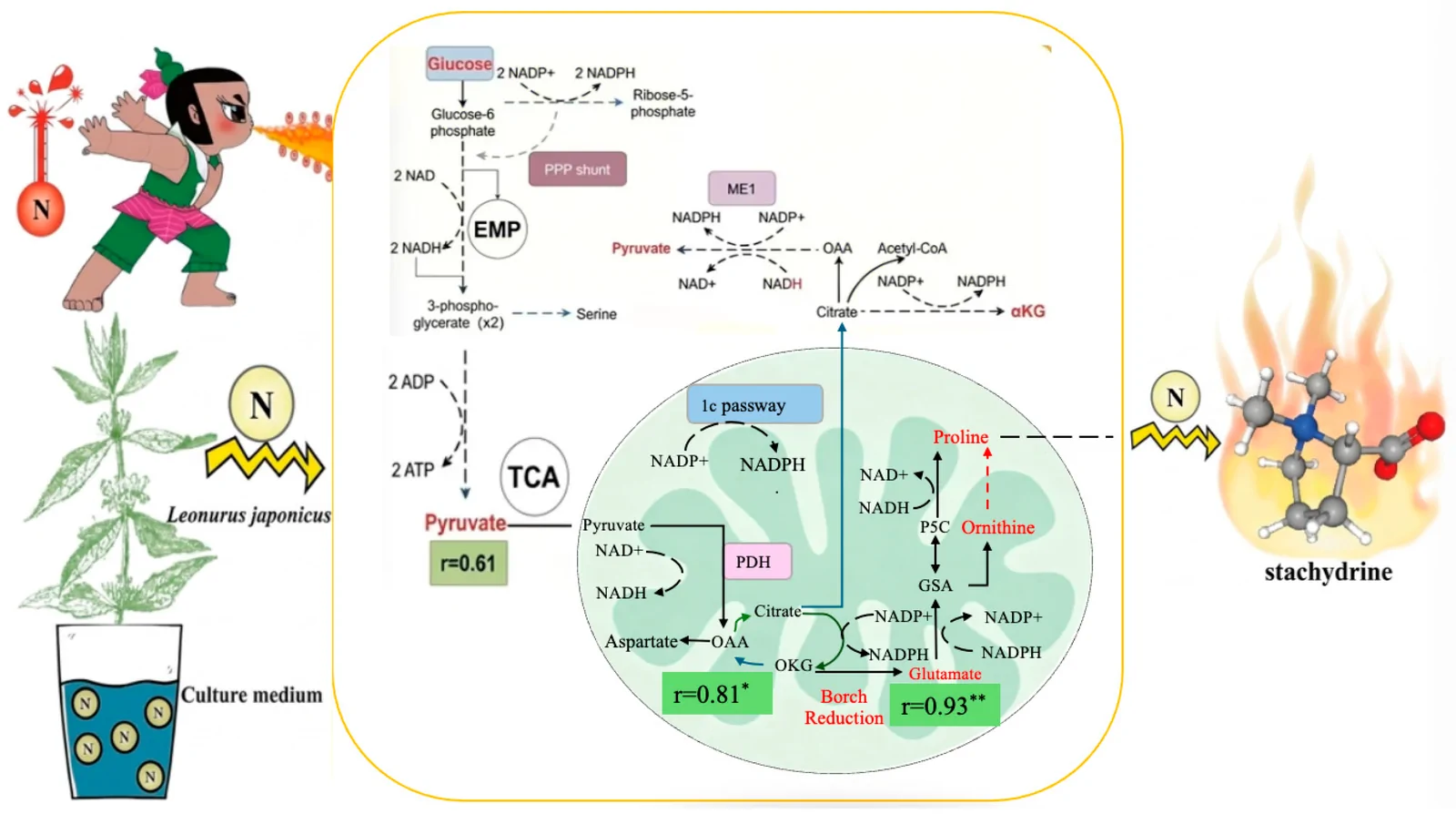
The mechanism of nitrogen involvement in the synthesis pathway of stachydrine in *L. japonicus*. Mechanisms of N involvement in the synthesis of stachydrine in *L. japonicus*. Spitfire represents photosynthesis; compounds labeled in red represent key intermediates involved in N. R in the green box represents the correlation of stachydrine with the substance, * represents significant correlation (*p* < 0.05), and ** represents highly significant correlation (*p* < 0.01). EMP represents the glycolytic pathway, TCA represents the tricarboxylic acid cycle, and αKG stands for α-ketoglutarate.

Stachydrine, identified as the primary active constituent in *L. japonicus*, belongs to the nitropyrrolidine alkaloid class [44]. In the medicinal plant *L. japonicus*, proline and N-methylproline undergo a continuous methylation reaction under the action of MT37 to form stachydrine [45]. There are two ways to synthesize proline in the body: one is through the perglutamic acid pathway, and the other is through the ornithine pathway. Among them, the glutamate pathway is a key determinant of osmotic stress and nitrogen deficiency. The ornithine pathway is dominant in the case of sufficient nitrogen [46,47,48]. Based on its chemical structure and existing literature, we hypothesize that ornithine serves as a precursor material [49,50]. Based on the above research, we propose a hypothesis: exogenous application of nitrogen can promote the switch of the proline synthesis pathway in Leonurus artemisia, preferentially synthesize proline through the ornithine pathway, increase the level of endogenous proline, and then provide sufficient precursors for the MT37-mediated methylation reaction, and finally promote the biosynthesis of stachydrine. The accumulation of stachydrine appears to be intricately linked to the availability of the substrate (ornithine). Exogenous nitrogen input is presumed to play a role in stachydrine formation from ornithine by participating in amino acid biosynthesis, providing a nitrogen source, and modulating the alkaloid’s synthetic pathway (Figure 3). We illustrated the mechanism of nitrogen involvement in the synthesis pathway of stachydrine in *L. japonicus* (Figure 4). Through correlation analysis, we determined the correlation coefficients between intermediate products and end products, confirming the necessity of nitrogen for the quality formation of *L. japonicus*. While we observed an N-stimulated increase in ornithine, a precursor to proline and stachydrine, the proposed involvement of the ornithine-proline pathway and MT37-mediated methylation is a mechanistic model inferred from these correlations. Direct assays of ODC activity, proline content, and MT37 expression are needed to confirm this pathway. Regrettably, our study, while confirming the necessity of a suitable nitrogen source for ornithine and stachydrine synthesis, did not delve into the detailed demonstration of intermediate enzymes like ODC and stachydrine precursors. Future research endeavors should aim to provide a more in-depth understanding and accurate substantiation of this proposed mechanism.

## 4. Materials and Methods

### 4.1. Experimental Site and Materials

The experiments were conducted at Beijing Miyun Traditional Chinese Medicine Plantation. The seeds of *L. japonicus* were procured from a market in Anguo City, Hebei Province. Following experimental testing, the germination rate was determined to be 90%, the germination potential was 38.6%, and the average germination time was 5 days. Subsequently, seedlings of *L. japonicus* were utilized in the experiments.

### 4.2. Experimental Design and Field Management

Experiments were executed using a completely randomized block design. Initially, seedlings were transplanted into a 1/4 concentration of nutrient solution, and after 3 days, they were switched to a complete nutrient solution. Seven N treatments were applied, denoted as N0, N1, N2, N3, N4, N5, and N6, corresponding to concentrations of 0, 2.5, 15, 35, 55, 75, and 95 mmol·L^−1^, respectively.

The nitrogen (N) concentration gradient (0, 2.5, 15, 35, 55, 75, and 95 mmol·L^−1^) was designed based on preliminary experiments and previous studies on *L. japonicus* hydroponic cultivation [51]. The lowest concentration (0 mmol·L^−1^) served as the N-free control to establish baseline growth and stachydrine synthesis under N deficiency. The concentration of 15 mmol·L^−1^ was selected as the reference point because previous research identified it as the optimal N supply for *L. japonicus* growth and alkaloid accumulation. Concentrations of 35 and 55 mmol·L^−1^ were included to evaluate the effects of supra-optimal N supply on primary and secondary metabolism, while 75 and 95 mmol·L^−1^ were designed to induce N toxicity stress, allowing us to observe the full spectrum of plant responses from deficiency to toxicity and to identify the critical threshold for N-induced growth inhibition. The nutrient solution was renewed every 3 days throughout the 90-day experimental period. The total N supplied per treatment over the entire cultivation period was calculated as follows: total N (mmol per plant) = N concentration (mmol·L^−1^) × solution volume (L per container) × number of replacements. For example, the 15 mmol·L^−1^ treatment received a total of 15 × V × 30 = 450 V mmol N per plant over 90 days (where V is the volume of nutrient solution per container in liters).

The nutrient solution, formulated based on the deficiency nutrient solution outlined in “Modern Plant Physiology” edited by the Shanghai Institute of Plant Physiology, Chinese Academy of Sciences, consisted of KH_2_PO_4_, 0.02 Fe-EDTA, 0.5 × 10^−2^ H_3_BO_3_, 0.7 × 10^−2^ MnSO_4_·H_2_O, 0.3 × 10^−3^ CuSO_4_.5H_2_O, 0.77 × 10^−3^ ZnSO_4_·7H_2_O, and 0.55 × 10^−3^ NaMoO_4_·2H_2_O. In nitrogen-deficient and low-nitrogen treatments, Ca(NO_3_)_2_·4H_2_O and KNO_3_ were replaced with the same number of moles of CaCl_2_ and KCl. The high-nitrogen gradient was supplemented with the corresponding number of moles of CO(NH_2_)_2_. The nutrient solution underwent replacement every 3 days, and the pH was carefully maintained between 5 and 6 throughout the experiment [49].

### 4.3. Sample Collection and Measurement of Biomass

*Leonurus japonicus* seeds were germinated on medium under natural light. Seedlings were routinely cultured for two months with 1/10-strength Hoagland nutrient solution supplied every 20 days. Uniform seedlings were transplanted into 12 L plastic hydroponic containers (40 cm diameter, 16 cm height). Each container was covered with a perforated aluminum-plastic board (64 holes, 2 cm diameter, 6 cm spacing). Thirty seedlings were sponge-fixed upright per board, with three boards applied for different nitrogen treatments, resulting in 90 biological replicates in total; thirty seedlings constituted one independent experimental unit. Four holes on each board were equipped with glass tubes connected to a 60 W low-noise air compressor for continuous aeration. Plants were cultivated indoors with metal halide lamps providing 150~200 μmol·m^−2^·s^−1^ light intensity at the canopy level. The daytime and nighttime temperatures were controlled at 22~25 °C and 20~22 °C, respectively.

In the experiment, 15 plants (5 plants were selected from each independent experimental unit) were sampled at the 20th, 40th, 60th, and 90th days following nutrient solution treatment for the assessment of leaf length, leaf width, plant height, and aboveground fresh weight. For each nitrogen concentration treatment, three independent hydroponic containers (each 12 L) were used as biological replicates. At each sampling time (20, 40, 60, 90 days), 5 plants were randomly harvested from each container and pooled, giving a total of 15 plants per treatment per time point (n = 3 biological replicates, each replicate being the pooled sample from 5 plants).

### 4.4. Determination of the Pyruvic Acid, α-Ketoglutarate, and Amino Acid Content

To determine the pyruvic acid and α-ketoglutarate content, preparing the sample, 0.5 g of fresh *L. japonicus* sample was weighed, and 10 mL of 0.65 mol/L HCl (containing 0.1 mmol/L EDTA-2Na) and a small amount of quartz sand were added for thorough grinding. Subsequently, 2.5 mL of H_2_O was added to create a homogenate. The homogenate was then transferred to a 10 mL centrifuge tube and subjected to heating in a water bath at 80 °C for 10 min (with intermittent shaking 2~3 times). Following this, the sample was placed in an ice bath for 5 min and subsequently centrifuged (8500 r/min, 10 min). The supernatant was collected into a 1.5 mL EP tube. To the collected supernatant, sequentially, 175 µL of 0.2 mol/L NaOH (with 80 µL added to the EP tube as a control), 280 µL of 10 mmol/L phenylhydrazine hydrochloride, and 245 µL of 0.1 mol/L phosphate buffer (pH 7.0) were added. The reaction proceeded for 20 min at 26 °C. The resulting mixture was then subjected to 0.45 μm microporous membrane filtration. For chromatographic analysis, the mobile phase consisted of A:B in a ratio of 80:20 (A: phosphate buffer 13 mmol/L KH_2_PO_4_, 1 mmol/L K_2_HPO_4_, pH 6.0; B: 100% methanol). The analytical column utilized was a Diamonsil C18 column (4.6 nm × 250 nm, 5 μm) with ultraviolet (UV) detection at a wavelength of 324 nm. The injection volume was 10 µL, and the flow rate was set at 1.5 mL/min. A mixed standard sample containing 40 µmol·L^−1^ pyruvic acid and α-ketoglutarate was prepared for calibration purposes.

To determine the amino acid content, approximately 40 mg of *L. japonicus* powder was accurately weighed and subjected to hydrolysis using a 6 mol/L HCl solution at 110 °C for 22 h. Upon completion of the hydrolysis, the volume was adjusted to 10 mL and filtered through a 0.45 µm membrane. The HCl was removed by rotary evaporation, the residue was dissolved in distilled water, and then 2 mL from the 10 mL solution was injected into the autosampling bottle for analysis. A Hitachi L-8500 amino acid analyzer equipped with a Hitachi 2622 SC analytical column and a buffer flow rate of 0.4 mL/min was employed for the measurement. Standards for amino acids were obtained from a mixture of 20 amino acids provided by Ajinomoto Company, Tokyo, Japan.

### 4.5. Determination of the Content of Stachydrine and Total Alkaloids

The harvested *L. japonicus* samples were dried in an oven at 55 °C, pulverized into fine powder, and sieved through a 60-mesh sieve.

For stachydrine extraction, 1.0 g of the dried powder was accurately weighed and extracted with 20 mL of 75% ethanol under ultrasonication at 40 °C for 30 min. The extraction was repeated twice, and the combined supernatants were filtered and evaporated to dryness. The residue was redissolved in 10 mL of methanol for analysis. Stachydrine content was determined by thin-layer chromatography (TLC) scanning following the method of Zhang et al. [49], using stachydrine reference standard (purity ≥ 98%, Sigma-Aldrich, St. Louis, MO, USA). The mobile phase consisted of n-butanol–glacial acetic acid–water (4:1:1), and detection was performed at λ = 510 nm after spraying with Dragendorff’s reagent. The standard curve was Y = 7.7318X − 13.733 (R^2^ = 0.9999), with a linear range of 8.5–340 μg·mL^−1^.

Method validation: The precision was assessed by five consecutive measurements of the same reference standard solution, yielding an RSD of 0.42% (n = 5). The stability test showed that the sample solution was stable within 4 h, with an RSD of 0.83% (n = 5). The repeatability, determined by analyzing five independently prepared sample solutions from the same batch, gave a mean stachydrine content of 1.21% (RSD = 2.23%, n = 5).

For total alkaloid determination, the dried powder (0.5 g) was extracted with 10 mL of 1% HCl in 70% ethanol under ultrasonication at 50 °C for 40 min. The extract was filtered and adjusted to pH 9–10 with ammonia water, then partitioned three times with chloroform. The organic phases were pooled and evaporated to dryness. The residue was redissolved in 5 mL of 0.1 M HCl, and total alkaloid content was measured by residual colorimetry using Reinecke’s salt (ammonium reineckate) at λ = 525 nm [method reference]. The standard curve was Y = 0.0246X + 0.0462 (R^2^ = 0.9998), with a linear range of 3.5–460 μg·mL^−1^.

Method validation: The precision, determined by five consecutive measurements of the same reference solution, yielded an RSD of 0.22% (n = 5). The stability test showed that the colorimetric reaction remained stable within 120 min, with an RSD of 0.83% (n = 5). The repeatability, determined by analyzing five independently prepared samples, gave an RSD of 1.98% (n = 5).

### 4.6. Statistical Analysis

Statistical analyses and correlation analyses were carried out using Microsoft Excel 2016 and SPSS v26.0 (SPSS Inc., Chicago, IL, USA), and the obtained results were presented as mean ± standard deviation (SD). The main effects were determined using one-way analysis of variance (ANOVA), followed by Fisher’s protected least significant difference (LSD) test. Graphical representations in the manuscript were generated using Adobe Illustrator 2021 and GraphPad Prism 10.5.0.

## 5. Conclusions

We indicate a correlation with the hypothesis regarding the indispensable role of N in the quality formation of *L. japonicus* through hydroponic experiments and provide initial insights into the mechanism of nitrogen’s involvement in stachydrine synthesis. Specifically, the application of appropriate concentrations of N (≤35 mmol/L) was found to regulate the TCA cycle and assimilate amino acid metabolic pathways. Consequently, this regulation led to the increased accumulation of organic acids, such as pyruvate and α-ketoglutarate, enhancing the primary metabolic pathway of amino acid synthesis. This enhancement facilitated the accumulation of crucial precursors, glutamate and ornithine, for hydrastine synthesis. The increased accumulation further directed amino acid synthesis toward the alkaloid secondary metabolism, significantly boosting the accumulation of stachydrine. The strong correlation between N concentration and glutamate accumulation suggests that the reductive amination of α-ketoglutarate to glutamate may be a critical regulatory point under our experimental conditions. However, this hypothesis requires further validation through direct measurements of enzyme activities and gene expression. Our findings not only offer a valuable theoretical foundation for fertilization methods to enhance *L. japonicus* yield and active ingredient content but also underscore the importance of maintaining N concentrations within a healthy range to prevent plant death from nitrogen toxicity.

## Figures and Tables

**Table 1:** Effect of different N treatments on amino acid content of *L. japonicus* leaf under 60 days.

Amino Acids mg/100mg	Nitrogen Application Level/(mmol/L)						
	0	2.5	15	35	55	75	95
**Cysteosulfonic**	0.000	0.005	0.018	0.034	0.031	0.032	0.034
**Aspartic**	0.088	0.111	0.120	0.153	0.186	0.244	0.429
**Threonine**	0.047	0.046	0.033	0.057	0.058	0.061	0.065
**Serine**	0.045	0.042	0.045	0.063	0.064	0.072	0.082
**Glutamate**	0.102	0.129	0.140	0.241	0.304	0.360	0.527
**Glycine**	0.057	0.059	0.081	0.079	0.076	0.077	0.085
**Alanine**	0.062	0.064	0.079	0.084	0.079	0.089	0.096
**Cystine**	0.009	0.003	0.003	0.005	0.004	0.005	0.007
**Valine**	0.060	0.055	0.084	0.076	0.076	0.078	0.080
**Methionine**	0.017	0.005	0.013	0.007	0.007	0.010	0.012
**Isoleucine**	0.049	0.052	0.068	0.059	0.058	0.058	0.063
**Leucine**	0.087	0.102	0.121	0.100	0.096	0.100	0.106
**Tyrosine**	0.041	0.040	0.011	0.024	0.025	0.030	0.043
**Phenylalanine**	0.058	0.051	0.084	0.075	0.071	0.072	0.077
**Gamma-aminobutyric**	0.016	0.018	0.025	0.028	0.025	0.028	0.036
**Ornithine**	0.003	0.005	0.005	0.006	0.006	0.009	0.018
**Lysine**	0.066	0.081	0.090	0.091	0.082	0.084	0.089
**Histidine**	0.030	0.035	0.036	0.037	0.034	0.039	0.044
**Argnine**	0.054	0.085	0.068	0.124	0.118	0.125	0.250
**Total**	0.924	1.073	1.193	1.435	1.504	1.576	2.303

## Data Availability

Data will be made available on request. Anyone who would like to request data from this study via email can contact Yan Zhang: zhangyan8669@126.com.

## Abbreviations

The following abbreviations are used in this manuscript:

TCA	Tricarboxylic acid cycle
ODC	Ornithine Decarboxylase
